# Retraction Note: TREEFINDER: a powerful graphical analysis environment for molecular phylogenetics

**DOI:** 10.1186/s12862-015-0513-z

**Published:** 2015-11-05

**Authors:** Gangolf Jobb, Arndt von Haeseler, Korbinian Strimmer

**Affiliations:** Department of Statistics, University of Munich, Ludwigstr. 33, D-80539 Munich, Germany; Department of Computer Science, University of Düsseldorf, Universitätsstr. 1, D-40225 Düsseldorf, Germany; John von Neumann Institute for Computing, Forschungszentrum Jülich, D-52425 Jülich, Germany

## Retraction

The editors of *BMC Evolutionary Biology* retract this article [1] due to the decision by the corresponding author, Gangolf Jobb, to change the license to the software described in the article. The software is no longer available to all scientists wishing to use it in certain territories. This breaches the journal’s editorial policy on software availability [2] which has been in effect since the time of publication. The other authors of the article, Arndt von Haeseler and Korbinian Strimmer, have no control over the licensing of the software and support the retraction of this article.
